# Loss of TNFRSF21 induces cisplatin sensitivity in lung adenocarcinoma

**DOI:** 10.32604/or.2024.050182

**Published:** 2025-02-28

**Authors:** DAIEN ZHOU, HAOYANG YUAN, YIWEI HU, CHUXU WANG, SA GE, KOUFENG SHAO, HONGYING WANG, XIAOFENG TIAN, HAIBO HU

**Affiliations:** 1 Department of Thoracic Surgery, The Affiliated Huai’an Hospital of Xuzhou Medical University, The Second People’s Hospital of Huai’an, Huai’an, 223001, China; 2 Medical Faculty, Kunming University of Science and Technology, Kunming, 650000, China; 3 The First School of Clinical Medicine, Nanjing Medical University, Nanjing, 210000, China; 4 Department of Oncology, Huai’an Chuzhou Hospital of Traditional Chinese Medicine, Zhongda Hospital Group Hospital Affiliated to Southeast University, Huai’an, 223001, China

**Keywords:** TNFRSF21, Lung cancer, Cancer stem cells (CSCs), Cisplatin sensitivity

## Abstract

**Background:**

Despite the identification of numerous therapeutic targets in lung cancer, achieving significant efficacy has been challenging. TNFRSF21 plays an important role in various cancers. We investigated the function of TNFRSF21 in lung adenocarcinoma (LUAD).

**Methods:**

The prognostic value of TNFRSF21 expression in lung cancer was evaluated by the GEPIA and Kaplan-Meier Plotter databases. Lung cancer cell viability was assessed by the CCK8 assay. TNFRSF21 expression patterns in lung cancer tissues and cells were examined using RT-PCR assay. Tumor sphere growth was evaluated through tumor sphere formation assays. MtROS contents in lung cancer cells were observed through MitoSOX fluorescent assays.

**Result:**

TNFRSF21 was up-regulated in LUAD patients. TNFRSF21 induction was particularly notable in LUAD, especially in cancerous cells (A549, H1299, H460, and SPC-A1), compared to BEAS-2B cells. Additionally, TNFRSF21 was increased in cisplatin (DDP)-resistant LUAD cells. Loss of TNFRSF21 significantly inhibited LUAD cell growth. It was observed that forced expression of TNFRSF21 contributed to tumor cell proliferation and DDP resistance. The production of ROS was found to participate in the inhibitory effects on lung cancer stem cells (CSCs), with decreased TNFRSF21 restraining ROS contents. Collectively, these findings reveal that the downregulation of TNFRSF21 promotes ROS contents to restrain the lung CSC-like characteristics via modulation of CD44 and CD133.

**Conclusions:**

In conclusion, TNFRSF21 may act as a novel target for lung cancer chemotherapy, particularly for eradicating lung CSCs.

## Introduction

Lung cancer is classified into small cell lung cancer (SCLC) and non-small cell lung cancer (NSCLC) [1,2]. Recently, there has been a notable increase in LUAD cases, accompanied by consistently poor patient survival rates [3]. Recurrence and metastasis, especially post-surgical removal, stand as the primary reasons for the high prevalence of LUAD [4]. For many years, DDP has been used as chemotherapy across various cancers, including LUAD. However, while some individuals may not react to DDP, others, despite initially being responsive, may eventually develop resistance [5]. Cancer stem cells (CSCs) are the key drivers of tumor recurrence [6]. The progression and recurrence of various cancers, including LUAD, are significantly influenced by CSCs [7].

Recent studies increasingly indicate that CSCs share characteristics with stem cells [8,9]. Additionally, specific surface markers (Nanog, Oct4, Sox2, CD44, and CD133) are expressed by lung CSCs, identified in NSCLC cell lines and patients. Several marker genes including CD133, CD44, and CD90 are associated with CSCs in lung cancer [10]. CSCs significantly influence the progression and recurrence of various cancers, including LUAD [11]. Lung CSCs are isolated from NSCLC cells and patients [12]. Understanding the role of CSCs is crucial for comprehending tumor emergence and recurrence fully.

TNFRSF21 plays a vital role in regulating the immune system [13]. It is expressed in most human tissues and induces apoptosis through NF-κB and JNK activation when ectopically expressed in mammalian cells [14]. While it defends against infection, it also adversely affects the mammary gland [15]. Recent reports suggest TNFRSF21’s involvement in esophagus adenocarcinoma progression [16]. TNFRSF21 is upregulated in relapsed pulmonary carcinoid (PC) after surgical resection. However, TNFRSF21’s functional role in LUAD advancement remains unclear.

Using GEPIA analysis, we found increased TNFRSF21 expression correlating with poor prognosis. Additionally, inhibiting TNFRSF21 decreased lung cancer cell growth and improved sensitivity to DDP. Therefore, our goal is to investigate the intricate mechanisms by which TNFRSF21 regulates LUAD progression.

## Materials and Methods

### Ethical approval

The study was approval by Institutional Reviewer Board of The Affiliated Huai’an Hospital of Xuzhou Medical University (Approval number: HEYLL202315); and all animal experiments conducted at The Affiliated Huai’an Hospital of Xuzhou Medical University were approved by the Institutional Animal Care and Use Committee (Approval number: HEYLL202315).

### GEPIA and Kaplan-Meier Plotter database

The predictive significance of TNFRSF21 expression in human malignancies was assessed using the GEPIA and the Kaplan-Meier plotter database.

### Cell culture

A549, H1299, H460, SPC-A1, and BES-2B normal lung epithelial cells were sourced from ATCC. All cells were cultured in DMEM supplemented with 10% FBS. DDP was purchased from Sigma in St. Louis, Missouri, United States. A549/DDP and H1299/DDP cells were established following the procedure outlined in a previous study [17]. These DDP-resistant cell lines were maintained in 1 μg/mL DDP. All cells were incubated in a humidified environment with 5% CO_2_ at 37°C.

### Plasmids and shRNA transfection of TNFRSF21

The pcDNA3.1(+)-TNFRSF21 plasmid was constructed as follows: Sangon Biotech (Shanghai, China) synthesized the TNFRSF21 mRNA fragment, which was then incorporated into pcDNA3.1(+) vector (Invitrogen, Carlsbad, CA, USA). For cell transfection, lung cancer cells were exposed to lentivirus containing either TNFRSF21 shRNA or sh-control (Genechem, Shanghai, China). After 6 h, the medium was changed using a solution containing 1 μg/mL puromycin (Sigma, USA) for 2 days to obtain TNFRSF21 shRNA or sh-control cells.

### CCK8 assay

The resistance of DDP was assessed using a CCK8 kit (Beyotime, Haimen, China). Transfected cells were cultured in 96-well plates and treated with various concentrations of DDP. After treatment, 20 μL of CCK8 solution was added for 4 h, and cell viability was determined by measuring the absorbance at 450 nm.

### Formation of tumor spheres

The stem cell medium consisted of DMEM/F12, 2% B27, 20 ng/mL EGF, 10 ng/mL bFGF, 0.4% BSA, and 5 μg/mL insulin. Tumorspheres measuring 100 μm or larger were counted after 7 days. Tumorspheres were imaged using a ZOE™ Fluorescent Cell Imager (Bio-Rad, CA, USA), and their sizes were measured using ImageJ software.

### EdU assay

The EdU Cell Proliferation Kit (Beyotime) was utilized for the EdU assay. A549/DDP and H1299/DDP cells were seeded onto 12-well dishes. After a 2-h incubation with 10 μM EdU, the cells were fixed using 4% paraformaldehyde for 10 min. 0.3% Triton X-100 for another 10 min was used to permeabilize the cells. Subsequently, EdU was detected by performing a click reaction for 30 min. Nuclear Proliferating cells were observed using fluorescence microscopy.

### Colony formation assay

Lung cancer cells (400 per well) were seeded onto 60 mm diameter plates. After transfection, the growth medium was changed every 3 days. Colonies were fixed using methanol for 10 min and stained using 0.1% crystal violet solution for 10 min after a 2-week incubation period. Images of colonies were captured using a digital camera, and ImageJ software was used for colony quantification.

### Flow cytometry analysis of CD44, CD133 and ki-67 positive cells

Following transfection, cells were harvested and treated with APC-linked monoclonal CD44 antibody (Biolegend, San Diego, CA, USA, Cat#163604), PE-linked monoclonal CD133 antibody (Biolegend, Cat#372804), or PE-linked monoclonal Ki-67 antibody (Biolegend, Cat# 350504) for 30 min at 4°C. Live cells were labeled using live-dead BV421 (Biolegend, Cat# 423114). Flow cytometry (BD, USA) was used to test the proportion of cells positive for CD44, CD133, or Ki-67.

### Transwell assay

Cells were suspended at a concentration of 5 × 10^4^ cells per well (200 μL) and placed in the upper chambers (24-well migration chambers, 8.0 μm pore membrane, Corning) using serum-free medium. Matrigel (1:5; Corning, New York, NY, USA) was applied to the upper chambers to facilitate cell invasion, while cell migration was unaided. 800 μL medium with 10% FBS was added to the lower chambers. Non-migrated or non-invaded cells in the upper chambers were removed using cotton swabs. Methanol was used to fix the cells adhering to the lower membrane surface and they were stained by 0.1% crystal violet. Images were captured using a microscope in five randomly chosen microscopic fields.

### qRT-PCR

TRIzol reagent (Invitrogen) was used to obtain total RNA. cDNA synthesis was performed using a cDNA synthesis kit (Applied Biological Materials, Richmond, British Columbia, Canada). EvaGreen 2 × qPCR MasterMix was used with a LightCycler96 real-time detection system (Roche, Switzerland). Amplification included an initial step at 95°C for 5 min, followed by 40 cycles at 95°C for 15 s, optimal annealing temperature for 30 s, and 72°C for 60 s. Primer sequences for PCR were shown in Table 1. RAN expression levels were calculated using 2^−ΔΔCt^ method.

**Table 1:** Primers applied in real-time PCR

Genes	Forward (5′-3′)	Reverse (5′-3′)
GAPDH	GGAGCGAGATCCCTCCAAAAT	GGCTGTTGTCATACTTCTCATGG
TNFRSF21	ATTGGCACATACCGCCATGTT	GGCTTGTGTTGGTACAATGCTC
CD133	GCTGCTTGTGGAATAGACAGAATG	GAAGGACTCGTTGCTGGTGAAT
CD44	ACATCCTCACATCCAACACCTC	CCTCCTGAAGTGCTGCTCCT

### Tumor xenografts

5 × 10^6^ A549/DDP cells, either infected with TNFRSF21 shRNA or not, were injected subcutaneously in the right armpit of male nude mice (12 in total, 6 weeks old). Tumor size was measured every three days starting from day 14. After the experiment was completed, the mice were euthanized, and tumors were excised and weighed. Tumor tissue samples were collected for qRT-PCR, western blot analysis, or flow cytometry. BALB/c nude mice were obtained from the Animal Research Center at Nanjing Medical University. All animal experiments conducted at The Affiliated Huai’an Hospital of Xuzhou Medical University were approved by the Institutional Animal Care and Use Committee (Approval number: HEYLL202315).

### MDA, SOD, GSH, and ROS detection

After treatment, cells were collected. MDA and SOD levels in lung cancer cells were evaluated using MDA Detection Kit (#BC0020, Beyotime) and SOD Activity Detection Kit (#BC0175, Beyotime). GSH levels were assessed using the GSH Content Detection Kit from Jiancheng Biotech Co. in Nanjing, China. Additionally, the Reactive Oxygen Species Detection Kit (#S0033, Beyotime) was used. DCFH-DA (2′,7′-Dichlorodihydrofluorescein diacetate) was incubated with cells at 37°C for 20 min. Cells were then examined using a fluorescence microscope, and cellular ROS fluorescence intensity was quantified.

### Determination of apoptosis

Cell apoptosis was assessed by FITC Annexin V Apoptosis Detection Kit (BD, USA). In a six-well plate, 5 × 10^5^ cells were seeded and incubated at 37°C. Adherent cells were detached using trypsin-EDTA, then centrifuged, washed twice with PBS, and suspended in 1 × binding buffer. Subsequently, 100 μL of the cell suspension was transferred to a 5 mL culture tube and incubated in the dark for 30 min with 5 μL FITC Annexin V and 5 μL PI. An additional 400 microliters of binding buffer were added. Flow cytometry (BD, USA) was used to measure fluorescence intensity.

### MitoSOX fluorescent assay

MitoSOX Red Dye (Invitrogen; M36008) was used to test mitochondrial reactive oxygen species (mtROS). Cells were indicated with 5 μM MitoSOX Red Dye and incubated for 10 min. After staining, cells were washed with PBS and examined using a Nikon A1R confocal laser scanning microscope system (Nikon, Tokyo, Japan).

### Statistical analysis

GraphPad Prism 9.5 was used to do statistical analysis. Data were presented as mean ± standard deviation. One-way ANOVA was employed for comparing statistical analysis among multiple groups. Unpaired two-tailed Student’s *t*-test was used to compare statistical analysis between two groups, Statistical significance was considered at *p* < 0.05.

## Results

### TNFRSF21 is overexpressed in LUAD

**Figure 1:**
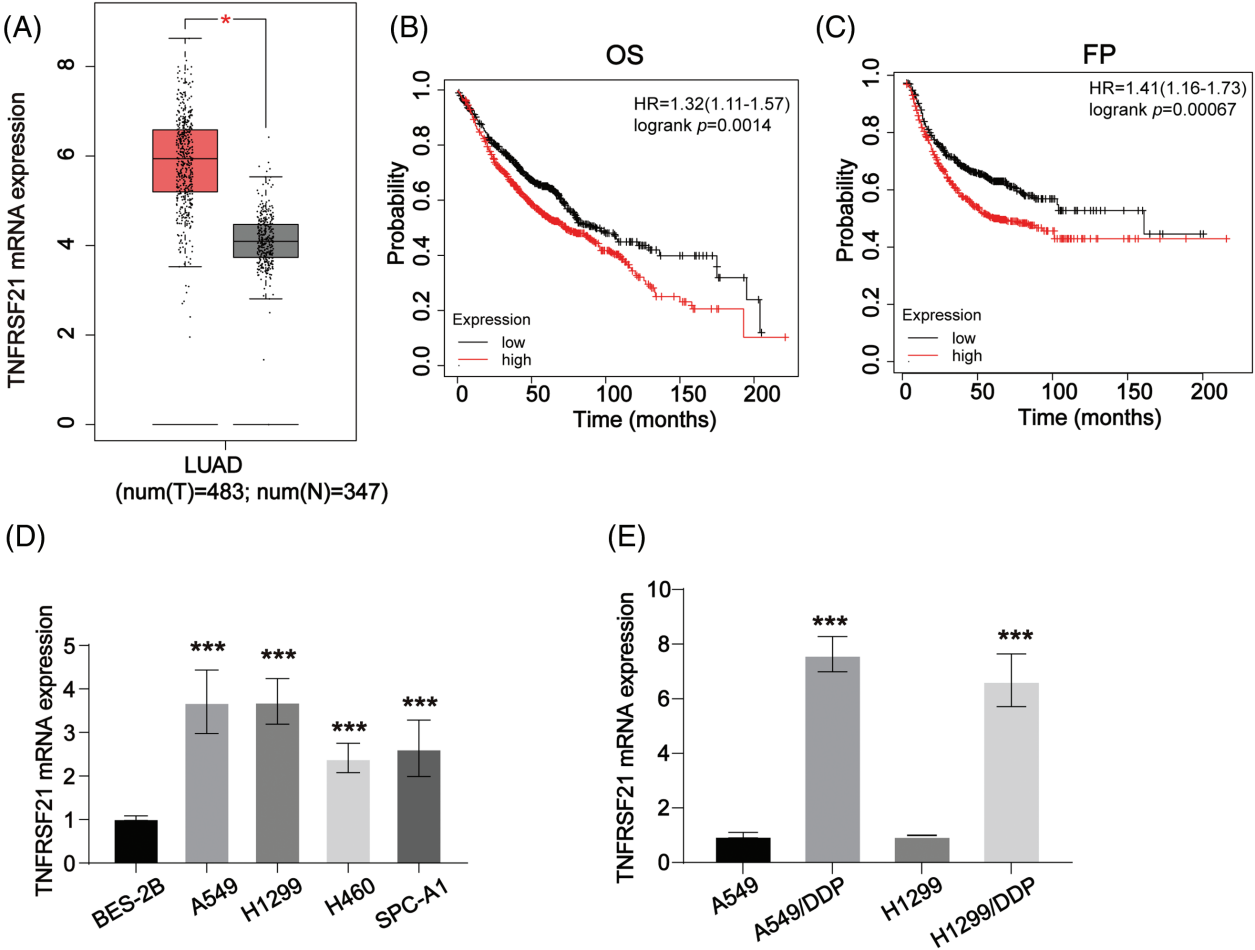
TNFRSF21 is increased in LUAD. (A) The overexpressed expression of TNFRSF21 in LUAD. The data were analyzed from GEPIA database. (B) Kaplan-Meier plotter displayed the OS status of LUAD patients. (C) Kaplan-Meier curve displayed the progression-free survival (FP) status of LUAD patients. (D) mRNA level of TNFRSF21 in LUAD cells (A549, H1299, H460, SPC-A1 cells) and normal cells (BES-2B cells). (E) The mRNA level of TNFRSF21 in LUAD/DDP resistant cells and the parental cells. *p < 0.05, ***p < 0.001. Three or more independent biological replicates were carried out.

We utilized the GEPIA database to evaluate TNFRSF21 expression in LUAD, revealing an elevation in LUAD tissues (Fig. 1A). Subsequently, Kaplan-Meier analysis (Fig. 1B,C) indicated that elevated TNFRSF21 levels correlated with unfavorable OS and FP in LUAD patients. Moreover, mRNA of TNFRSF21 was increased in A549, H1299, H460, and SPC-A1 cell, while BES-2B exhibited decreased TNFRSF21 expression (Fig. 1D). Additionally, Fig. 1E demonstrates higher TNFRSF21 levels in A549/DDP and H1299/DDP cells. Taken together, these findings suggest TNFRSF21’s potential as a promising prognostic biomarker for LUAD.

### Loss of TNFRSF21 represses the malignancy of LUAD

**Figure 2:**
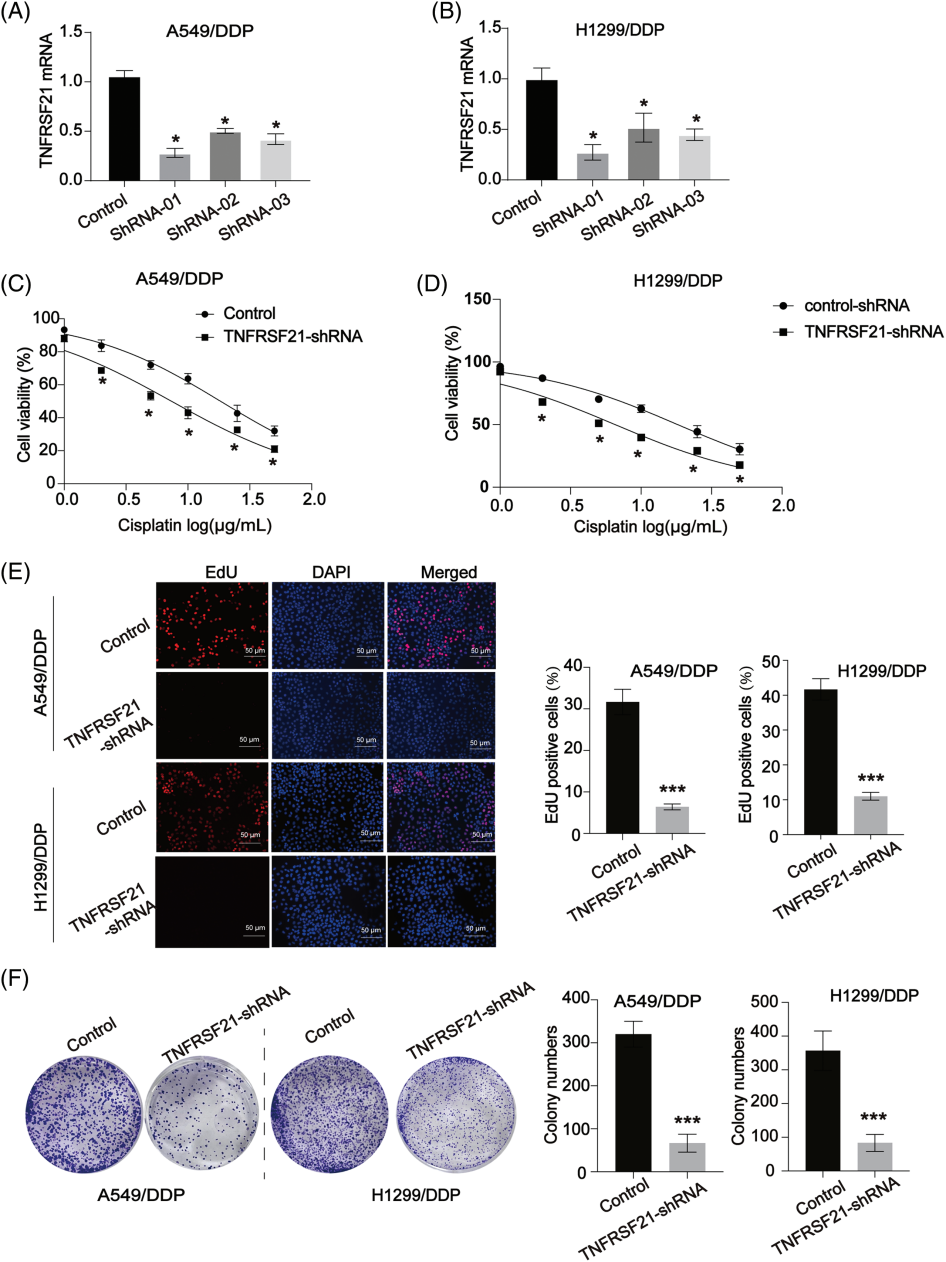
Loss of TNFRSF21 inhibits LUAD cell proliferation and induces DDP sensitivity. (A and B) mRNA expression of TNFRSF21 in A549 and H1299 cells infected by TNFRSF21 shRNA. (C and D) A549/DDP and H1299/DDP cells were treated with different concentrations of DDP for 24 h. CCK-8 assays were performed to examine cell viability. (E) Representative EdU results exhibited the proliferation of LUAD cells after loss of TNFRSF21. Scale bar = 50 μm. (F) Representative colony formation experiments showed the colony formation capacity of A549 and H1299 cells after decreasing TNFRSF21. *p < 0.05; ***p < 0.001. Three or more independent biological replicates were carried out.

Experiments aimed at reducing TNFRSF21 expression were conducted using A549/DDP and H1299/DDP cells as the experimental models. TNFRSF21 expression was efficiently reduced in A549/DDP and H1299/DDP cells by applying shRNA, with sh1 showing the highest knockdown efficacy and thus chosen for subsequent assays (Fig. 2A,B). Notably, decreased cell viability was observed in A549/DDP and H1299/DDP cells when exposed to various DDP doses (Fig. 2C,D). Furthermore, the impact of TNFRSF21 on LUAD cell proliferation was evaluated using the EdU assay and colony formation experiment, revealing that suppressing TNFRSF21 significantly hindered the proliferation of A549 and H1299 cells (Fig. 2E,F).

**Figure 3:**
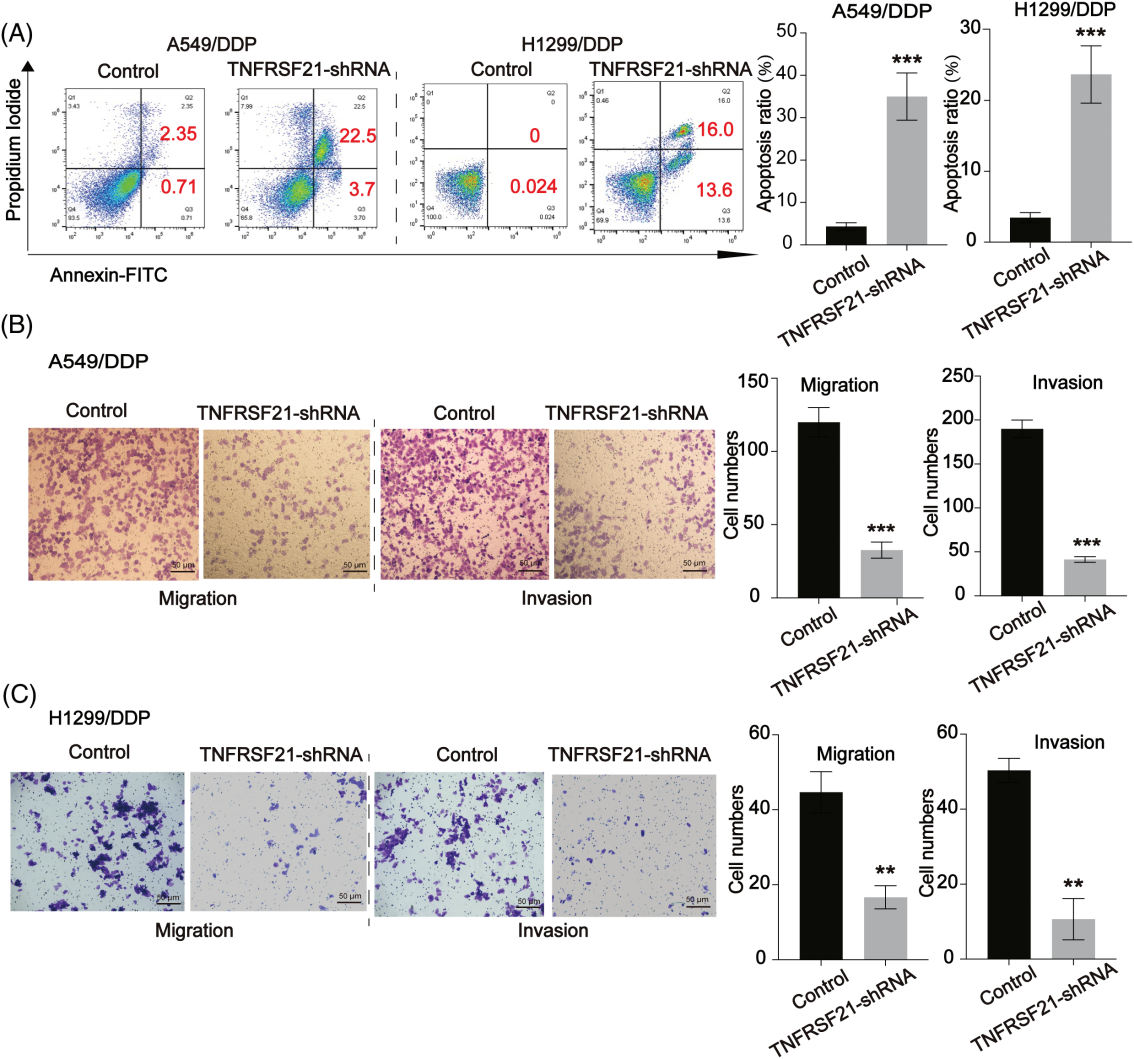
Loss of TNFRSF21 triggers LUAD cell apoptosis and represses cell migration and invasion. (A) Flow cytometry analysis indicated the apoptotic A549 and H1299 cells. (B and C) Migration and invasion ability of A549 and H1299 cells. Scale bar = 50 μm. **p < 0.01; ***p < 0.001. Three or more independent biological replicates were carried out.

Additionally, a cell colony formation assay demonstrated a down-regulation in the colony-forming ability upon TNFRSF21 depletion (Fig. 3A). TNFRSF21 shRNA treatment notably increased apoptosis in A549 and H1299 cells, as observed in the flow cytometry assay (Fig. 3A). Transwell assays were employed to investigate the effects of TNFRSF21 knockdown on the migration and invasion of LUAD cells. Suppression of TNFRSF21 led to a reduction in the migratory and invasive capacities of lung cancer cells (Fig. 3B,C), suggesting the involvement of TNFRSF21 in LUAD.

### The stemness of LUAD cells is enhanced by TNFRSF21

**Figure 4:**
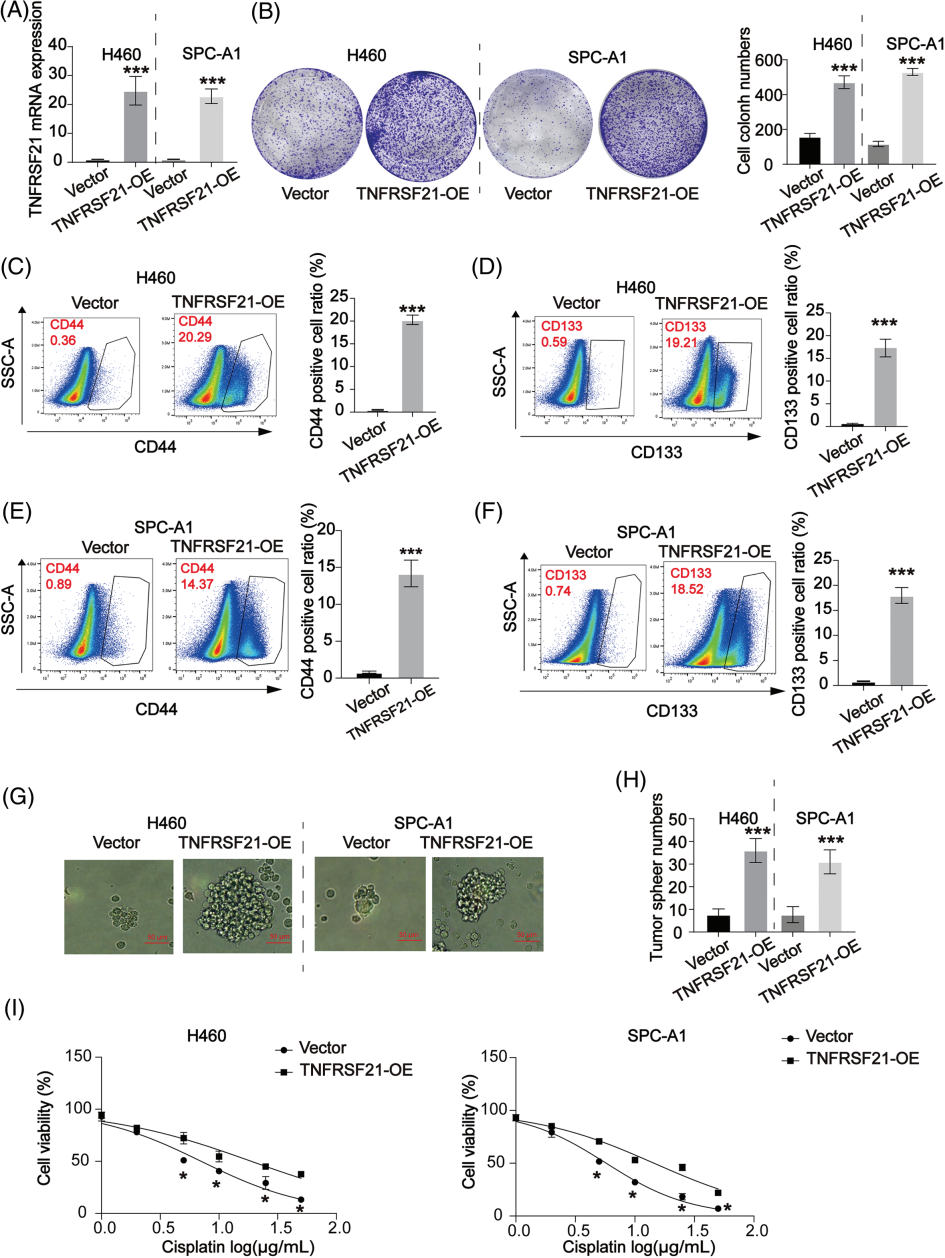
Overexpressed TNFRSF21 induced LUAD cell proliferation via activating LUAD cell stemness. (A) qPCR data confirmed TNFRSF21 mRNA expression in H460 and SPC-A1 cells transfected with TNFRSF21 overexpression plasmid. (B) Representative cell colony formation images of H460 and SPC-A1 cells overexpressing TNFRSF21. (C and D) Representative flow cytometry images demonstrate the percentage of CD44+ and CD133+ H460 cells following TNFRSF21 overexpression. (E and F) Representative flow cytometry images show CD44+ and CD133+ ratio of SPC-A1 cells after TNFRSF21 increase. (G and H) Representative tumor sphere images of H460 and SPC-A1 cells. Scale bar = 50 μm. (I) CCK-8 assays were performed to assess cell viability. *p < 0.05, ***p < 0.001. Three or more independent biological replicates were conducted.

To understand the role of TNFRSF21, we conducted overexpression experiments on H460 and SPC-A1 cells, which exhibit low levels of TNFRSF21 expression. Using shRNA, we downregulated the expression of TNFRSF21 in H460 and SPC-A1 cells. qPCR data in Fig. 4A confirms TNFRSF21mRNA expression in H460 and SPC-A1 cells transfected with the overexpression plasmid-pcDNA3.1-TNFRSF21. Enhanced expression of TNFRSF21 significantly increased the capacity of H460 and SPC-A1 cells to form colonies, as depicted in Fig. 4B. Flow cytometry results revealed a significant proportion of CD44 and CD133 positive cells in H460 and SPC-A1 cells with increased TNFRSF21 expression (Fig. 4C–F). Moreover, transfected cells showed enhanced ability to form tumor spheres upon TNFRSF21 overexpression, as shown in Fig. 4G,H. TNFRSF21 also reduced the DDP sensitivity of H460 and SPC-A1 cells as illustrated in Fig. 4I. These findings suggest that increased TNFRSF21 expression enhances the maintenance of CSCs.

### In LUAD, the presence of CSC-like markers is directly associated with TNFRSF21

**Figure 5:**
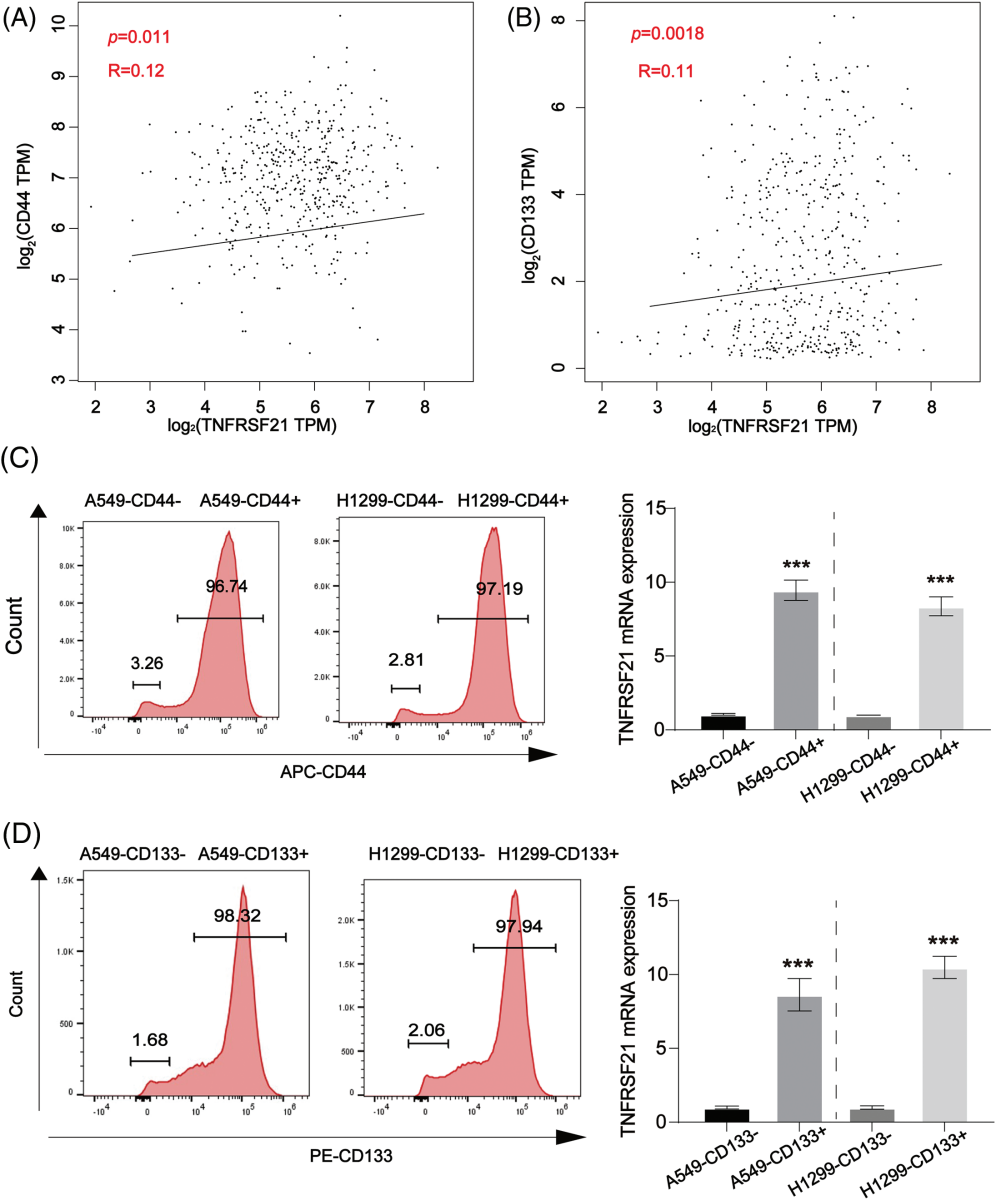
Positive correlation between CD44, CD133 and TNFRSF21 in LUAD tissues and LUAD/CSCs. (A) Correlation between CD44 and TNFRSF21 in LUAD tissues via consulting GEPIA database. (B) Correlation between CD133 and TNFRSF21 in LUAD tissues via consulting GEPIA database. (C) mRNA expression of TNFRSF21 tested in CD44-positive A549 and H1299 cells isolated by flow cytometry. (D) mRNA expression of TNFRSF21 tested in CD133-positive A549 and H1299 cells isolated by flow cytometry. ***p < 0.001. Three or more independent biological replicates were conducted.

In LUAD, GEPIA findings revealed a favorable association between the CSC-like indicators CD44 and CD133 and TNFRSF21, as depicted in Fig. 5A,B. Fig. 5C,D demonstrates a notable increase in TNFRSF21 mRNA expression within CD44/CD133 positive A549 and H460 cells compared to CD44/CD133 negative LUAD cells. These findings indicate a positive correlation between increased TNFRSF21 expression and the level of stemness in LUAD.

### Inhibition of TNFRSF21 activated oxidative stress in LUAD cells

**Figure 6:**
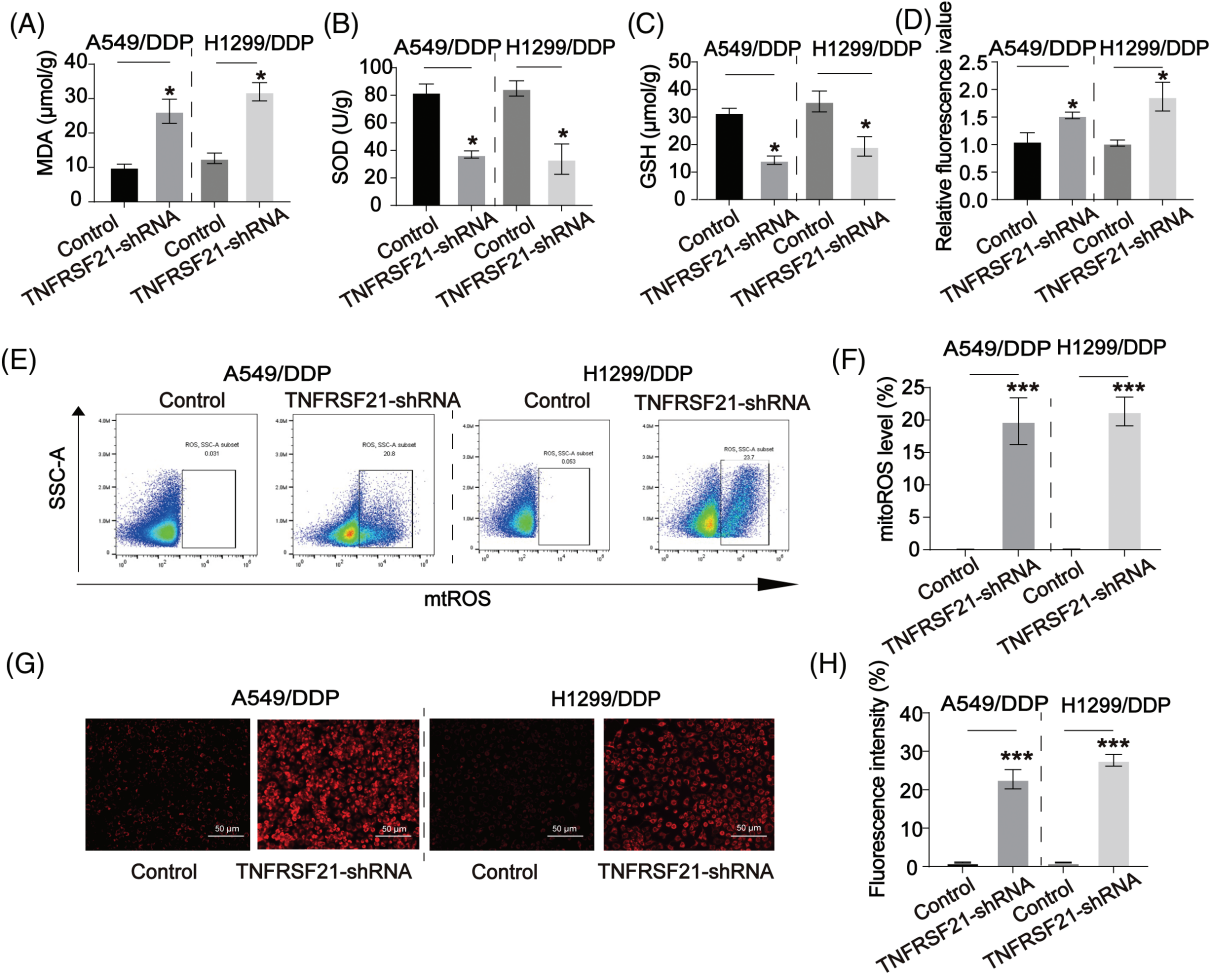
Downregulated TNFRSF21 activated ROS contents in LUAD/DDP resistant cells. (A) MDA contents in LUAD DDP cells. (B) SOD contents in LUAD DDP cells. (C) GSH contents in LUAD DDP cells. (D) ROS contents in LUAD DDP cells. (E and F) Flow cytometry results of MitoSOX staining in LUAD DDP cells. (G and H) MitoSOX staining showing ROS levels in LUAD DDP cells. Scale bar = 50 μm. *p < 0.05; ***p < 0.001. Three or more independent biological replicates were conducted.

ROS production plays a significant role in the inhibitory effects on lung CSCs. In Fig. 6A–D, it was observed that the use of TNFRSF21 shRNA resulted in a higher accumulation of MDA and ROS in DDP-resistant lung cancer cells compared to the control group. Furthermore, LUAD cells exhibited a decrease in the SOD and GSH. Moreover, we assessed the buildup of mitochondrial ROS by employing MitoSOX staining. This analysis unveiled that the depletion of TNFRSF21 triggered an elevation of mitochondrial ROS levels (Fig. 6E,F). Consistency in the staining of ROS levels was shown in DDP cells using MitoSOX (Fig. 6G,H). These findings suggest that the decrease of TNFRSF21 activates oxidative stress levels and mitochondrial ROS accumulation in LUAD cells.

### Decreased TNFRSF21 inhibits the xenograft tumor growth in vivo

**Figure 7:**
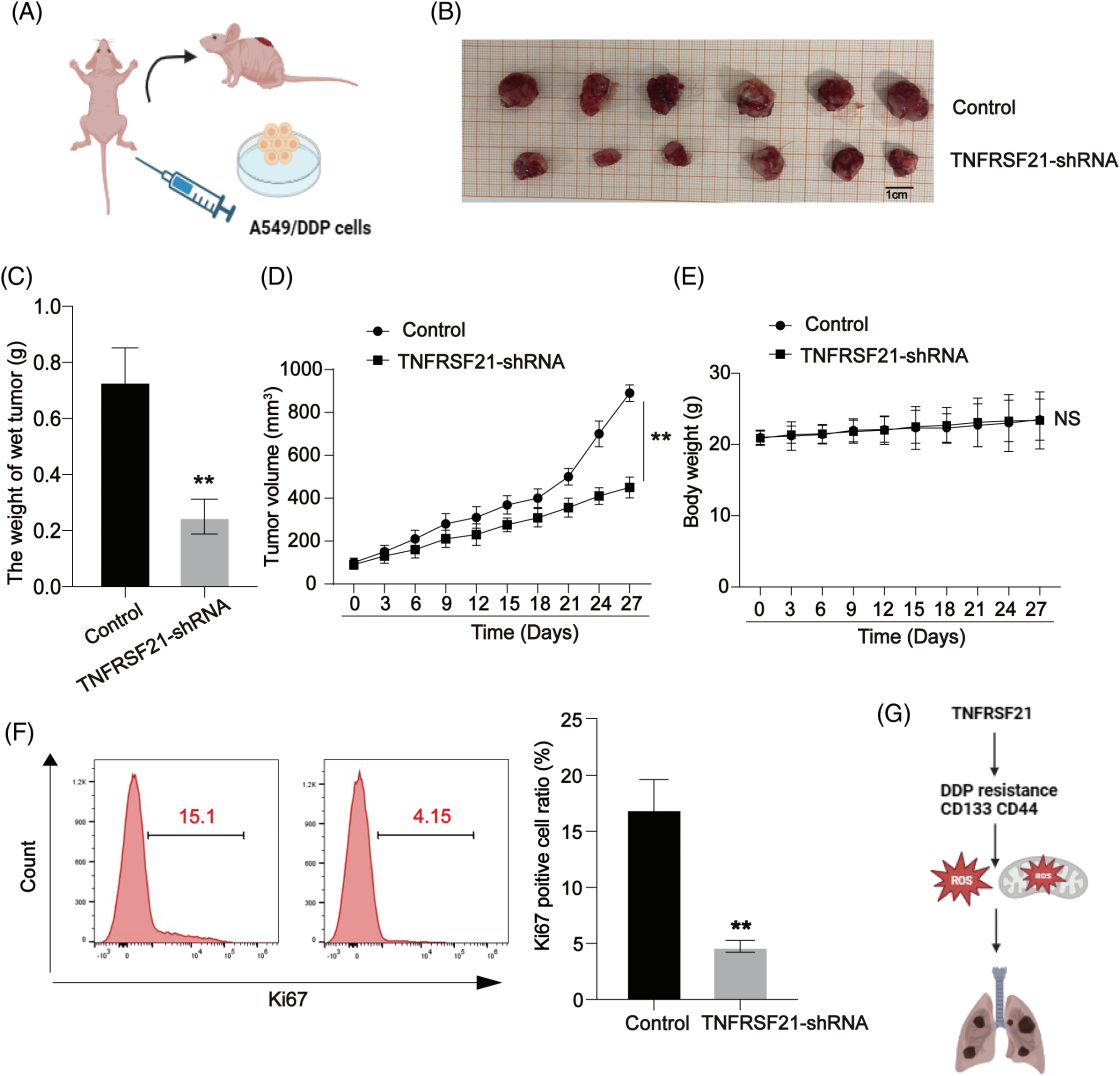
Decrease of TNFRSF21 reduces lung cancer growth *in vivo*. (A) A549/DDP cells were transfected with TNFRSF21 shRNA. Nude BALB/c mice were injected with transfected A549/DDP cells. (B) Tumor images upon sacrifice of the mice. (C) Tumor weights. (D) Tumor volumes calculated every 2 days with growth curve. (E) Body weight calculated every 2 days with growth curve. (F) Proliferation index Ki-67 in xenografts assayed using flow cytometry. (G) Overall research diagram. **p < 0.01. Three or more independent biological replicates were conducted.

In this investigation, a xenograft tumor mouse model of LUAD using A549/DDP cells allowed us to assess the impact of TNFRSF21 on the progression of lung carcinoma, as illustrated in Fig. 7A. Our study results revealed that mice injected with A549/DDP cells infected with TNFRSF21 exhibited reduced tumor sizes and decreased average tumor masses, as depicted in Fig. 7B,C. Furthermore, there was a gradual decrease in the rate of tumor growth over time, as shown in Fig. 7D. Fig. 7E demonstrates that the tumorigenicity was suppressed by the knockdown of TNFRSF21, further confirmed through flow cytometry analysis of Ki-67. These findings suggest that TNFRSF21 contributes to *in vivo* LUAD progression (Fig. 7F). Fig. 7G summarizes our overall research diagram.

## Discussion

Despite the utilization of various innovative treatment strategies for LUAD, mortality remains primarily attributed to resistance, recurrence, and metastasis. Chemotherapy resistance poses a substantial hindrance to clinical management. The prominence of CSCs has grown in various malignancies, demonstrating their significant involvement in tumor recurrence and drug resistance [18]. Resistance to chemotherapy presents a significant challenge in the clinical management of LUAD. CSCs have gained prominence across various malignancies, showing significant involvement in tumor recurrence and drug resistance [19]. They contribute to chemotherapy ineffectiveness and tumor recurrence [20,21]. However, the precise mechanisms underlying CSCs’ distinctive self-renewal remain incompletely understood [22,23]. The lack of efficient medications targeting CSCs may stem from the intricate mechanisms involved in their progression.

Our investigation focused on the fundamental factors contributing to LUAD CSCs advancement. We observed a significant elevation of TNFRSF21 in both LUAD patients and lung cancer cells. Elevated TNFRSF21 expression correlated with an unfavorable prognosis in lung cancer patients. Additionally, we created A549/DDP and H1299/DDP cells, noting a significant increase in TNFRSF21 expression in LUAD cells resistant to DDP. However, we did not collect lung cancer tissues. Obtaining clinical samples is essential for further validation in our future research.

Numerous research studies have documented the possible involvement of TNFRSF21 in various cancers [24]. TNFRSF21, linked to necroptosis, has been associated with skin cutaneous melanoma prognosis. Pancreatic adenocarcinoma shows a notable increase in TNFRSF21, and its absence provides significant survival benefits [25]. Furthermore, TNFRSF21 is significantly upregulated in TNBC compared to other subtypes. We found elevated levels of TNFRSF21 in both lung cancer tissue samples and cells. Suppression of TNFRSF21 significantly inhibited lung cancer cell progression, while inducing apoptosis. However, the detailed effect of TNFRSF21 in normal tissues requires further investigation.

The induction of ROS facilitates cancer growth, influencing cell proliferation, survival, and apoptosis. Cancer cells typically exhibit higher ROS levels compared to normal cells, crucial for maintaining the cancer phenotype [26,27]. In contrast, CSCs have lower ROS levels compared to cancer cells [28,29]. Recent studies indicate that mitochondrial stability actively influences CSC fate by regulating nuclear stemness gene expression and cell division outcomes [30]. In our investigation, we observed that excessive TNFRSF21 expression stimulated lung cancer stem cell characteristics by promoting CD133 and CD44 mRNA expression. Additionally, loss of TNFRSF21 induced ROS levels. However, the correlation between ROS levels and CSC self-renewal remains unverified. Our research findings provide insight into the fundamental processes TNFRSF21 supports in lung carcinoma CSCs.

## Conclusion

In summary, our study elucidates a mechanism whereby TNFRSF21 alters lung cancer cell fate by regulating CSC-like properties and ROS accumulation. Ectopic TNFRSF21 expression modulated cancer stem cell properties and sensitivity to DDP. The identification of TNFRSF21 in LUAD enhances our understanding of lung cancer’s fundamental biology, offering insights into managing chemoresistance.

## Data Availability

The data that support the findings of this study are available from the corresponding author upon reasonable request.
